# Progress in Psychiatry

**Published:** 1900-04-28

**Authors:** 


### Progress in Psychiatry.

(*Continued from page 53.*)

**Genealogical Studies.**—No more striking and accurate way of presenting this phase of the problem can be compared with the genealogical method as applied to families which exhibit the nervous degenerations alluded to. Some genealogical tables have appeared in preceding chapters (pages 212 and 327, Vol. XXVII), and the following may now be added:

Case A.—A watchmaker having recovered from an attack of insanity caused by the Revolution of 1789, finally committed suicide by poison. Later on his daughter became insane, and died of dementia. A son attempted suicide by striking a knife into his abdomen. Another of his children suffered from delusions of persecution, and refused food so that he died of starvation. A fifth became a chronic drunkard, and died on the wayside. The sixth, a daughter, who was of good health, married and gave birth to five children. Of these five, one, a boy, was an epileptic imbecile; the second, a girl, suffered from puerperal insanity and refused food; the third was an infant who refused to be suckled; and the fourth and fifth died of cerebral disease.[22]

Case B.—In a family studied by Berti,[32] and comprising four generations of about 80 individuals descended from an insane melancholiac, there were 10 who suffered from insanity nearly always melancholia, 19 who were neurotic, three with criminal tendencies, and three with special abilities.

Case C.—Already referred to, and published by Morel (page 327 *ante*).

Case D.—Reported by Legrain, and already recorded (page 327 *ante*).

Case E.—First generation: Man alcoholic. Second generation: Two sons, both tuberculous; a third son a drinker; the fourth, a daughter, who is an invalid with melancholic ideas. Her issue comprises the following (third generation): two children; one is an alcoholic lunatic, the other becomes a general paralytic, and leaves one child as follows (fourth generation): one child, dies of meningitis.[33]

The family of the "Jukes," studied by Dugdale,[34] is of great importance with regard to the light it throws on the problems of heredity, pauperism, crime, and insanity. The ancestor of the family was Max Juke (born between 1720 and 1740) a squatter. He was a man of hard-drinking habits, averse to steady work, and by turns hunter and fisher, who grew blind in old age. He left two sons, who married two girls out of a family of six sisters, some of whom were illegitimate, and three of whom had had illegitimate issue. "Of these six sisters, the descendants of five have been studied. The progeny of these five sisters has been traced with more or less exactness through five generations. . . . The number of descendants registered includes 540 individuals who are blood relations of the Jukes, and 169 who are related by marriage or cohabitation; in all, 709 persons of all ages. The aggregate of this lineage reaches, probably to 1,200 persons, but the dispersions that have occurred at different times have prevented the following up of many of the lateral branches. This vast family . . . has been, on the whole, a family of criminals and prostitutes, of vagabonds and paupers. Of all the men not 20 were skilled workmen, and 10 of these learnt their trade in prison; 180 received outdoor relief to the extent of an aggregate of 800 years; or, making allowances for the omissions in the records, 2,300 years. Of the 709 there were 76 criminals, committing 115 offences. The average of prostitution among the marriageable women down to the sixth generation was 52·4 per cent.; the normal average has been estimated at 1·66 per cent." Dugdale estimates that the total cost of maintenance (in prisons and penitentiaries, outdoor relief, hospital or workhouse, &c.), of the Jukes family on the State of New York amounted to over a million dollars.

Insanity in twins is of interest as regards the light it throws on one aspect of heredity. Euphrat[25] gives an account of twin females, aged 40, who were both the subjects of hallucinations of the senses, and suffered from delusional ideas. MacDowall has published an instructive case,[26] in which twin boys, aged 17, the issue of a vicious, morally insane woman, were subjects of imbecility, and developed in time delusions of persecution. Other interesting examples have been recorded by Savage[27] and others on this interesting subject. Very interesting investigations on the ancestry, collaterals, and descendants of epileptics and of general paralytics have been recently published by several writers,[28] and will be referred to in their proper places.

**Pathogenesis of Insanity.**—The insanities have, like other diseases which affect the organism, their special modes of origin, of development, and of termination. In every case the central organ which is affected in insanity is the brain, and in a narrower sense the localisation of mental disease may be regarded as appertaining to various centres and tracts in the brain in varying proportions. The child comes into the world with a brain which has but one-third the mass (quantitatively) of the full-grown brain, and with but little of the complexity of organisation which (qualitatively) also constitutes the adult brain. And the whole period of childhood and youth constitutes a period of development—quantitative and qualitative—for the nervous system in general, and brain in particular. For after birth variety begins in the sentient and respondent life of the child, and such variations imply corresponding variations impressed upon and organised within its nervous system. The training and education of the nervous system begins. The new-born child can perform some movements well; the nervous mechanisms for these have been almost fully-formed in the bulbo-spinal centres at birth, and require but the incidence of appropriate stimuli to call responses forth almost in perfection. Among such may be mentioned the acts of breathing, of crying, of sucking, of swallowing, and of grasping with the hands and fingers an object placed within the hand. To these may be added the special reflex movements of sneezing anu coughing. The child in its somnolescent and vegetative stage of life is capable of these acts, and it is of interest to know that in idiots of the lowest grade, as well as in dements reduced to profound depths of mental dissolution, such acts as the above may persist almost intact. A later stage of cerebral evolution is marked by the appearance of instinctive acts, and at a still later date, that is, after the sixth month of infancy, definitely imitative acts and movements appear; and together with these there develop the corresponding emotions of child-life. "With the first impulses to imitation those to significant vocalisation are born. Emulation rapidly ensues, pugnacity follows in its train. Fear of definite objects (partly instinctive, but partly also acquired in association with painful or disagreeable experiences) is also developed early. . . . Play, curiosity, acquisitiveness, and even greed all manifest themselves with the first two years of life."[29] An arrest of special cerebral and mental evolution at these levels of development (while the rest of the nervous system and body continue in their growth) marks the various types of imbecility and of congenital feeblemindedness.

The co-operation of language—itself a product of cerebral mechanism, and having its starting points in the auditory centres—at this stage marks a period of great importance. For this method of response to, and of communication with, other individuals of the human species attains to a great psychological and economic value. For it is obvious that language is useful in proportion to (*a*) its symbolistic and representative power, (*b*) its comprehensiveness, (*c*) the economy of labour and time it affords in expressing and communicating ideas, and (*d*) its ability to lead to higher and more complex cerebral processes. Words and expressions become convenient and economic symbols for the varied and multiform items and groups of experiences. The evolution of numbers and of numerical processes of computation constitute a further interesting phase in the development of language, and the reduction of both to writing marks a yet higher grade of evolution. It will be shown in subsequent chapters how these higher cerebral processes are affected in different forms of insanity.

The self-assertiveness and aggressive of the growing ego wherever it comes into contact and into antagonism with nature is a factor of great importance. For with the increase in extent and variety of the environment with which the child comes into contact, with every obstruction encountered and overcome, the empirical ego grows in clearness, in integration of consciousness, and in uncritical aggressiveness. The rapidly-growing forebrain begins to take part as the predominant partner of psychical life, while inherited instincts and propensities now seek for more determinate expression as the mechanisms of such expression (otherwise known as the kinæsthetic centres) advance in development. Between these instincts and impulses on the one hand and the conditions and circumstances imposed by nature and by society on the other there is continual stress and conflict. The child thus learns to adapt itself to nature (intellectual progress), and to realise and fulfil the relationships and duties of life towards its fellows (moral progress). In pre-adolescent development the child is still a being largely wrapped up in its instincts of self-preservation and aggressiveness; it is, in short, a wholly egoistic being, greedy and grasping, dominated by the immediate and the present, a parasite with all its instincts converging towards the animal self. The sexual life is as yet undeveloped. In many forms of imbecility and of congenital mental and moral defect, as well as in certain neuropaths and psychopaths, the above characters and peculiarities play an important part.[30]

Lastly, we come to the stages which comprise puberty and adolescence—the years of sexual unfolding which comprise the decade between 15 and 25. Anomalies and troubles of evolution, and mental derangements, occurring at this period have a special stamp of their own. Disturbances of a hysterical, mystico-religious, and erotic type mark this period of life in many, and the insanities of this period are similarly characterised. A tendency to precocious dementia may also develope, combined with stuperose and cataleptic disturbances.

[22] Lombroso, Man of Genius: London. [23] Legrain, loc. cit. [24] Dugdale, the Jukes passim. [25] Zeitschr. für Psycholog., H. 2 and 3, 1887. [26] Jour. of Mental Science, 1884. [27] Jour. of Mental Science, 1883. [28] Wahl. These de Paris, 1899; Bechet, These de Paris, 1899. [29] Lloyd Andriezen, Jour. of Mental Science, April, 1899, p. 271. [30] Ibid., April, 1899, passim.

